# Mediating Role of Time Spent on Social Media in the Relationship Between Flourishing and Problematic Internet Use Among Indian Emerging Adults

**DOI:** 10.34172/jrhs.12747

**Published:** 2026-08-15

**Authors:** Sona Varghese, Abdul Raffie Naik

**Affiliations:** ^1^School of Social Sciences and Humanities (VISH), VIT-AP University, Vijaywada, India

**Keywords:** Flourishing, Problematic internet use, Social media, Emerging adults, Well-being

## Abstract

**Introduction:**

The rapid expansion of internet access and social media use has transformed communication and social interaction globally; however, it has also raised concerns regarding negative psychological outcomes. Emerging adults, often described as "digital natives," may be particularly vulnerable to these effects. This study aimed to investigate the associations between flourishing, Problematic Internet Use (PIU), and time spent on social media among emerging adults.

**Study Design:**

A cross-sectional online survey.

**Methods:**

The cross sectional study was conducted from. February to April 2025 with 1,044 participants (59.9% men, 40.1% women; mean age=18.85, SD=1.38). Participants completed self-report measures assessing flourishing using the Mental Health Continuum-Short Form, PIU using the PIU Questionnaire, and daily time spent on social media. Mediation analysis was conducted using PROCESS macro Model 4 while controlling for age and gender to examine whether time spent on social media mediated the association between flourishing and PIU.

**Results:**

Greater time spent on social media was associated with lower flourishing and well-being scores, including emotional, social, and psychological well-being dimensions. In contrast, PIU and its subcomponents (obsession, control disorder, and neglect) increased significantly with higher social media use. Mediation analysis indicated that time spent on social media significantly mediated the negative relationship between flourishing and PIU (indirect effect=-0.047, 95% CI: -0.068, -0.028, *P*<0.001).

**Conclusion:**

These findings are consistent with the displacement hypothesis and the I-PACE model, suggesting that lower flourishing is associated with increased social media engagement, which may contribute to the development of PIU. The results highlight the importance of integrating flourishing-enhancing interventions and digital wellness strategies to reduce PIU risks among digitally immersed emerging adults.

## Introduction

 As of 2025, approximately 67.9% of the global population, representing nearly 5.56 billion individuals, uses the internet. Among these users, 63.9% (5.24 billion people) actively engage with social media platforms, which are broadly defined as websites or applications that facilitate social communication and interaction.^1^ People use these platforms for a wide range of purposes, ranging from satisfying basic social needs to knowledge seeking and gambling.^2-4^ Although social media offers benefits such as enhanced connectivity and educational resources,^5^ it also presents risks.^6-8^

 The growing prevalence of internet use has heightened concerns regarding its problematic forms. Problematic Internet Use (PIU), also referred to as problematic internet usage, is considered a form of technological addiction characterized by excessive interaction with digital systems. PIU involves maladaptive patterns of use, excessive preoccupation with internet activities, and diminished self-regulation, often resulting in adverse effects on personal, professional, and social functioning.^9-10^ It may also function as an overarching construct encompassing various maladaptive online behaviors, particularly when available evidence is insufficient to support a clinical diagnosis of addiction.^11^

 Previous research has demonstrated that PIU is significantly correlated with a range of adverse psychological conditions such as anxiety, depression, and loneliness, as well as risky behaviors such as substance abuse, impulsivity, diminished self-esteem, and suicidal ideation.^9,12-13^ Although the relationship between PIU and poor mental health is sometimes bidirectional, existing evidence indicates that the causal pathway more frequently originates from PIU, leading to subsequent mental health challenges. Moreover, predisposing factors such as emotional dysregulation, impulsivity, fear of missing out (FoMO), and maladaptive coping mechanisms further increase the risk for PIU.^14-16^ In this research, PIU refers to maladaptive patterns of internet use regardless of the specific platform or purpose, with time spent on social media conceptualized as a behavioral indicator rather than a synonym for PIU.

 Multiple theoretical approaches have attempted to explain the link between PIU and mental health. The displacement hypothesis suggests that internet use displaces beneficial activities (e.g., sleep, and face-to-face interaction). Uses and Gratifications Theory (UGT) posits that individuals actively select media to satisfy needs, often including escapism, implying that poor mental health may predict increased use. Person-Affect-Cognition-Execution (I-PACE model) highlights the dynamic interaction between personal characteristics, affective states, cognitive processes, and executive functions, suggesting that PIU develops as a maladaptive coping pattern for managing life stressors.^17-23^

 Beyond the mere absence of pathology, psychological well-being is increasingly conceptualized through the construct of flourishing. Flourishing represents a state of optimal human functioning that integrates both hedonic and eudaimonic dimensions of well-being. Prominent frameworks include Seligman’s PERMA model^24^, Keyes’ mental health continuum^25^, and broader multidimensional models incorporating physical health, character, and financial security.^26-27^ Flourishing is empirically linked to improved mental and physical health, resilience, and success in occupational and educational contexts.^27-29^

 Young adults in their twenties constitute nearly one-third of total social media users.^6^ Adolescents and young adults are generally more sensitive to both the benefits and harms of social media platforms compared to older cohorts.^30^ Generation Z, comprising individuals born after 1995, is the first generation raised entirely in the digital age. They are often referred to as “digital natives,” “the Facebook generation,” or “the online generation”.^2^ This group occupies the developmental stage known as emerging adulthood (approximately ages 18-29), a period marked by significant life transitions.^31^ Increased digital exposure during this stage can exert a significant impact on their well-being.

 There is substantial body of empirical research that has examined the relationship between PIU and well-being; however, most studies have focused on adolescents and have yielded ambiguous or inconclusive findings. Flourishing is critically relevant in this context as it represents a comprehensive indicator of optimal mental health and psychosocial functioning. Given that emerging adults are uniquely vulnerable due to their developmental stage and extensive exposure to digital environments, this study sought to address these gaps by investigating the associations between flourishing, PIU, and time spent on social media in this population.

## Methods

###  Participants

 The initial participant pool consisted of 1,082 respondents who met the following inclusion criteria: (a) being 18 years of age or older, (b) being 29 years of age or younger, (c) owning a smartphone or laptop, and (d) having access to Wi-Fi or an internet connection.

 Ownership of a smartphone or laptop and access to Wi-Fi or the internet were considered essential because the primary objective of the study was to examine the frequency of social media use and its association with flourishing and problematic internet use. Participants without regular access to such devices or internet connectivity would have limited or no opportunities to engage in online activities, making their behavioral patterns unsuitable for investigating the constructs under investigation.

 Data collection was conducted between February 2025 and April 2025. The final sample consisted of 1,044 participants, including 625 men (59%) and 419 women (40.1%), with a mean age of 18.85 years (SD = 1.38). Based on self-reported socioeconomic status, participants were distributed across five strata: lower class (4.5%), lower-middle class (5.9%), middle class (41.7%), upper-middle class (23.4%), and upper class (24.5%). In terms of family structure, most participants belonged to nuclear families (68.2%), followed by joint families (22.3%), extended families (3.2%), single-parent families (3.9%), grandparent-headed families (2.2%), and stepfamilies (0.2%). Participants were recruited from urban (62.0%), suburban (14.8%), and rural (23.2%) areas.

###  Instruments

 The survey included the following instruments, along with sociodemographic variables such as age, gender, and daily breakfast consumption

###  The Mental Health Continuum-Short Form 

 The Mental Health Continuum-Short Form (MHC-SF) was developed by Keyes (2009). It is a 14-item instrument designed to assess positive mental health across three dimensions: emotional well-being, social well-being, and psychological well-being. Response are rated on a 6-point Likert scale ranging from “never” (1) to “every day” (6), with higher scores indicating greater levels of flourishing. The MHC-SF has demonstrated strong psychometric properties with high internal consistency for the total scale (α = .89-.95) and acceptable test-retest reliability (r = .65-.68 over 3-9 months). Confirmatory factor analyses support the three-factor structure comprising emotional, psychological, and social well-being dimensions, with measurement invariance across demographic groups. The instrument also demonstrates strong convergent validity through positive correlations with well-being measures (e.g., r = .66-.86 with the WHO-5 Well-Being Index) and good discriminant validity through negative correlations with mental illness indicators (r = -.33 to -.78), thereby supporting the two-continua model of mental health. Furthermore, cross-cultural validation studies conducted in multiple countries have confirmed the scale’s robust psychometric properties and broad international applicability.^32^

###  Problematic Internet Usage Questionnaire 

 The PIUQ is an 18-item self-report instrument designed by Pramod and Raju (2011) to assess pathological behaviors and psychological issues related to internet use.^33^ This tool is an Indian adaptation of the original PIUQ developed by Demetrovics et al (2008),^34^ maintaining a similar three-factor structure of the parent instrument. The questionnaire measures PIU across three primary dimensions: control issues, neglect, and obsession. Items are rated on a 5-point Likert scale, with total scores ranging from 18 to 90 points. Reliability evidence indicates strong internal consistency, with a reported Cronbach’s alpha of.87 for the total scale. Validity was established through concurrent validation procedures, demonstrating a strong correlation coefficient of r = .82 with related scales. The instrument comprises three distinct subscales: Obsession, Neglect, and Control Disorder, with a clinical cut-off score of 41 or above indicating that an individual is at risk for PIU. The questionnaire has been widely applied to identify patterns of internet usage across various research, educational, and clinical settings, showing satisfactory psychometric properties for assessing problematic internet behaviors among young adults in Indian populations.^33^

###  Procedure 

 The present study employed a cross-sectional research design to address the primary research objectives. To ensure rapid and efficient data collection from accessible individuals, a convenience sampling method was adopted. This enabled the researcher to collect data rapidly from accessible participants under time and resource constraints. The sample size was determined *a priori* using G*Power (version 3.1.9.7) to ensure adequate statistical power for multiple regression analyses examining the associations between flourishing, time spent on social media, and PIU in emerging adults. Power calculations targeted the detection of small to medium effect sizes (f^2^ = 0.02-0.15, equivalent to R^2^ = 0.02-0.13) in multiple regression models incorporating flourishing, social media, and PIU. Power analysis indicated that a minimum sample of 550-650 participants was required to achieve 80% statistical power at a significance level of α = .05 for regression models incorporating three to five predictor variables. The target sample size was subsequently increased to 1,082 respondents to account for potential data attrition and incomplete responses, as well as to enhance the statistical precision necessary for detecting the hypothesized associations between study variables. The final sample of 1,044 participants exceeded the minimum power requirements, yielding robust statistical power (> 90%) for detecting small effect sizes and enhancing the generalizability of the findings within the emerging adult population.

 The field survey was administered via QR codes linked to an online survey platform, which were displayed in classrooms to minimize paper usage, consistent with an environmentally friendly approach. Additionally, the survey link was shared across various social media platforms to reach a wider audience. The frequency of daily social media use was assessed with the item: “How many hours do you spend on various social media platforms such as Instagram, YouTube, and so on each day?” with responses categorized as less than 1 hour, 1–5 hours, and more than 5 hours. This classification reflects light, moderate, and heavy usage patterns, respectively. Utilizing a categorical duration format improves recall reliability and practicality. Instead of requiring participants to recall and provide exact hours and minutes, which is a task that is prone to error due to the fragmented and habitual nature of social media use, the categorical format allows respondents to select meaningful, distinct usage brackets that align with typical daily routines.

 Participants completed the questionnaire in English, as it served as the primary shared language among the linguistically diverse respondent pool. The average time required to complete the questionnaire was approximately 15 minutes.

###  Data Analysis

 Data cleaning, processing, and analysis were conducted using IBM SPSS Statistics (version 21.0) and the PROCESS macro. Descriptive statistics, including means, standard deviations, and percentage distributions, were calculated. The normality of the data was assessed using skewness and kurtosis values; following the criteria established by Kim for sample sizes exceeding 300, skewness values greater than 2 and kurtosis values greater than 7 were utilized as thresholds for substantial non-normality.^35^

 The researcher used the PROCESS macro (Model 4) to assess the mediating role of time spent on social media in the relationship between flourishing (predictor) and PIU (outcome). The model was estimated using a bootstrapping method with 5,000 samples and a 95% confidence interval. To ensure participant confidentiality, the data that support the findings of this study are available from the corresponding author, A.R.N, upon reasonable request.

###  Ethics

 The research was conducted in accordance with the Declaration of Helsinki and relevant legal and ethical standards appropriate to the local cultural context. The study protocol received formal ethical approval from the University Institutional Review Board (VIT-AP/IECI2025-26/007). All participants were informed about the nature and purpose of the study and were assured of their privacy, confidentiality, and right to withdraw from the study at any time. Written informed consent was obtained prior to questionnaire completion. No financial compensation or incentives were offered for participation.

## Results

Table 1 presents the mean scores and standard deviations for emotional well-being, social well-being, psychological well-being, flourishing, obsession, control disorder, neglect, and PIU across three groups defined by daily time spent on social media: less than 1 hour, 1–5 hours, and more than 5 hours. The data reveal a clear pattern: as daily time spent on social media increases, emotional, social, and psychological well-being, as well as flourishing, steadily decline, whereas PIU and its subcomponents (i.e., obsession, control disorder, and neglect) increase. Participants spending less than one hour daily on social media reported the highest levels of emotional well-being (M = 8.50, SD = 3.75), social well-being (M = 9.93, SD = 5.62), psychological well-being (M = 18.16, SD = 6.36), and flourishing (M = 36.59, SD = 13.26), as well as the lowest PIU scores (M = 35.52, SD = 10.83). In contrast, those who used social media for more than five hours per day reported the lowest emotional well-being (M = 6.19, SD = 4.18), social well-being (M = 8.64, SD = 6.14), psychological well-being (M = 12.77, SD = 7.20), and flourishing (M = 27.59, SD = 14.71), alongside the highest PIU scores (M = 52.17, SD = 15.26). The *P*-values indicate that the differences among the groups are statistically significant.

**Table 1:** The Mean Scores and Standard Deviations for Flourishing, Its Dimensions, PIU, and Its Dimensions Across Three Groups Defined by Daily Time Spent on Social Media

**Variables**	**<1 hour, n=205**		**1–5 hours, n=775**		**>5 hours, n=64**		***P*** **-value**
	**Mean**	**SD**	**Mean**	**SD**	**Mean**	**SD**	
Emotional Well-being	8.50	3.75	7.74	3.58	6.19	4.18	.001
Social Well-being	9.93	5.62	8.92	5.39	8.64	6.14	.050
Psychological Well-being	18.16	6.36	15.41	15.41	12.77	7.20	.001
Flourishing	36.59	13.26	32.06	13.07	27.59	14.71	.001
Obsession	12.13	4.01	15.28	4.56	18.09	5.77	.001
Control Disorder	12.03	4.52	13.74	5.05	17.56	5.66	.001
Neglect	11.36	3.79	13.83	4.57	16.52	5.30	.001
PIU	35.52	10.83	43.86	12.73	52.17	15.26	.001

*Note.* PIU: Problematic internet usage; SD: Standard deviation.

 According to Figure 1 and Table 2, the mediation analysis revealed that flourishing had a significant direct negative association with PIU (β = -0.121, 95% CI: -0.1787, -0.0641). Time spent on social media significantly mediated this relationship, with a significant indirect effect (*ab*= -0.047, 95% CI: -0.0678, -0.0282). Specifically, higher flourishing predicted less time spent on social media (β = -0.006, 95% CI: -0.0082, -0.0038), while greater time spent on social media was, in turn, associated with higher PIU (β = 7.781, 95% CI: 6.2147, 9.3484).

**Figure 1:**
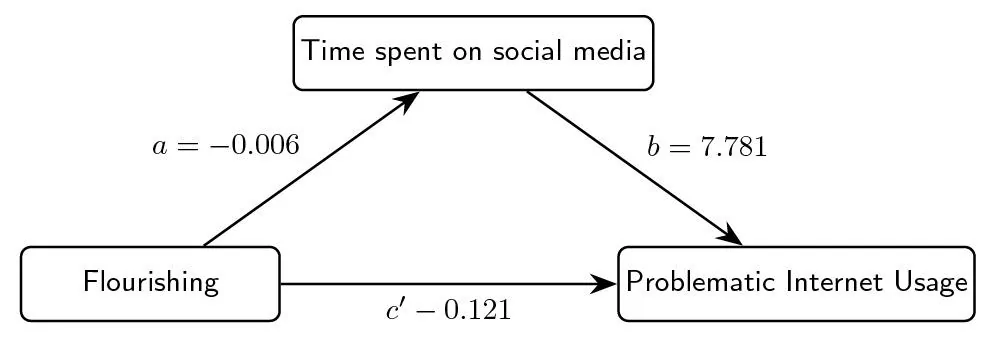
Mediation Analysis Results
*Note. *PIU: Problematic internet use. Time spent on social media significantly mediated the relationship between flourishing and PIU (mediation effect: -0.006 Í 7.781 = ‒0.047, *P* < 0.001)

**Table 2:** Mediation of TS in the Relationship Between Flourishing and PIU

**Path**	**Effect (b)**	**β**	**SE**	**t**	***P***	**LLCI**	**ULCI**
F → TS (a)	-0.006	-0.1648	0.0011	5.3949	0.001	-0.0082	-0.0038
TS → PIU (b)	7.781	0.2888	0.7985	9.7451	0.001	6.2147	9.3484
F → PIU (c')	-0.121	-.01232	0.0292	4.1560	0.001	-0.1787	-0.0641
Indirect Effect (ab)	-0.047	-0.0476	0.0102	-	-	0.0678	0.0282

*Note.* TS: Time spent on social media; F: Flourishing; PIU: Problematic internet use; SE: Standard error; LLCI: Lower-level confidence interval; ULCI: Upper-level confidence interval. Bootstrap sample size = 5000. All path coefficients are unstandardized.

## Discussion

 The findings of this study provide evidence supporting the hypotheses and reveal a significant relationship between time spent on social media, flourishing, and PIU among emerging adults. The results indicate that as time spent on social media platforms increases, indicators of well-being decline, while symptoms of PIU increase.

 The observed relationships in the present study support various theoretical perspectives. The displacement hypothesis states that time spent online displaces restorative offline activities such as sleep, hobbies, or in-person social interaction, thereby contributing to reduced well-being.^17^ Consistent with this view, this study found that heavier daily social media use was linked to markedly lower flourishing scores, suggesting that time spent on social media may displace offline activities, which could negatively impact the PERMA^24^ dimensions of positive emotions, engagement, relationships, meaning, and accomplishment.

 UGT and the I-PACE model also help contextualize these findings, particularly the marked increase in PIU and its subcomponents (obsession, control disorder, neglect), alongside decreased flourishing and well-being scores.^18,23^ These frameworks suggest that individuals, particularly those experiencing life stressors or emotional distress, may use social media for distraction or as a coping mechanism.^22,36^ Emerging adulthood is a period of significant transition and stress, making this demographic particularly vulnerable.^31^ Although social media may initially be used to alleviate negative feelings, this behavior can evolve into a compulsive cycle in which individuals lose control over their usage, leading to the development of PIU.

 The mediation analysis further clarifies this dynamic. The significant indirect effect indicates that the frequency of social media use is a critical pathway through which low flourishing is associated with PIU. Individuals with lower flourishing are more likely to engage in frequent social media use, and this high level of engagement is linked to the development of unhealthy digital usage patterns. Casale et al demonstrated that lower psychological well-being, characterized by elevated anxiety and loneliness, correlates with an increased risk of PIU.^37^ Similarly, a high frequency and duration of internet use have been identified as primary risk indicators for PIU and have been directly linked to diminished mental health, reduced sleep quality, and lower overall well-being.^38-40^ Thus, the findings of the current research are consistent with existing literature and support the notion that PIU is not merely an indicator of poor mental health but may also be a consequence of using social media as a maladaptive response to preexisting vulnerabilities.

 The integration of flourishing into this research provides a broader and more tailored interpretation of the outcomes. Traditional deficit-focused models often emphasize symptom reduction, which may fail to create a long-term impact in reducing the target behavior. The findings suggest the need for interventions from a holistic perspective, especially those focusing on proactive factors such as flourishing rather than merely on symptom reduction. Flourishing encapsulates both hedonic and eudaimonic dimensions of wellness.^25,27^ The strong inverse associations between flourishing and PIU observed in this sample imply that reduced well-being may increase the vulnerability of emerging adults to develop maladaptive coping strategies. Moreover, flourishing is linked to resilience and adaptive functioning across educational, occupational, and interpersonal contexts.^28,41-43^ Thus, interventions targeting flourishing, rather than merely reducing PIU, could be more effective in supporting emerging adults’ digital well-being. For example, positive psychology intervention techniques, such as gratitude journaling and acts-of-kindness exercises, may be useful.

 The findings have substantial practical implications across multiple domains and warrant attention from practitioners, educators, and policymakers working with emerging adults. Clinically, assessment protocols should systematically incorporate digital usage patterns when evaluating mental health, as these patterns may indicate declining well-being. Therapeutic interventions should adopt a dual approach that simultaneously addresses underlying vulnerabilities through positive psychology interventions aimed at enhancing flourishing, alongside cognitive-behavioral strategies designed to reduce maladaptive coping patterns. Since flourishing represents a comprehensive well-being construct encompassing happiness, meaning, purpose, physical and mental health, character, close social relationships, and financial stability that extends beyond traditional deficit-focused measures, interventions based on this framework may be particularly beneficial for emerging adults. Educational institutions should integrate digital wellness components into campus mental health initiatives and develop prevention programs that promote healthy usage patterns before problematic behaviors emerge. From a policy perspective, these results necessitate platform design modifications that incorporate natural stopping points and meaningful usage feedback. Future research should prioritize longitudinal studies to establish causal relationships, investigations of individual difference factors, intervention-effectiveness studies, and cross-cultural validation studies to develop more effective culturally adapted intervention approaches.

 The present study has a few limitations. First, it relied on self-report measures, which may have introduced social desirability bias. Second, the use of non-probability sampling might limit the generalizability of the findings. Third, the cross-sectional research design restricts the ability to establish causal relationships among the study variables; therefore, future studies should employ longitudinal and experimental designs to clarify the directionality of these relationships. Fourth, although the sample included emerging adults aged 18–29, the representation of individuals aged 18–25 and those aged 25–29 was uneven. Future research should ensure more balanced representation across the full 18–29 age range. Fifth, the measurement of social media use via a single self-report item with broad response categories represents a significant methodological limitation. This approach is susceptible to recall bias and social desirability bias and lacks precision regarding the nature of usage, such as active versus passive engagement. Future studies should employ more robust measures, such as experience sampling methods or objective tracking tools, to capture social media use more accurately. Additionally, since emerging adulthood and flourishing are culturally sensitive constructs, cross-country comparisons using nationally representative samples are needed. Finally, key potential confounders, such as preexisting mental health conditions and personality factors, were not controlled for, which may have biased the estimates. Future research should include these variables as covariates.

**Highlights**

- higher frequency of social media use among emerging adults was associated with lower levels of flourishing.
- The risk of Problematic Internet Use (PIU) was significantly higher among individuals with more frequent social media use.
- The study included a large sample of 1,044 emerging adults.

## Conclusion

 The present study provides empirical evidence for the mediating role of social media use in the relationship between flourishing and problematic internet use (PIU) among emerging adults. The results indicate that lower flourishing is associated with greater social media engagement, which, in turn, is associated with maladaptive usage patterns and heightened PIU. Although causal directions cannot be inferred from this cross-sectional study, these findings are consistent with the notion that PIU often develops as a secondary response to decreased well-being, thereby expanding theoretical support for the displacement hypothesis, UGT, and the I-PACE model. The study highlights flourishing not merely as the absence of psychopathology but as a comprehensive protective construct encompassing emotional, psychological, and social functioning. Consequently, interventions designed to cultivate flourishing, including meaning-making interventions, positive emotion regulation, and relationship-building strategies, may hold promise for mitigating digital overuse and its associated psychological risks.

 From a practical standpoint, these findings call for multidisciplinary responses. Clinicians should evaluate digital behavior patterns alongside mental health assessments, while educators and institutions should implement digital wellness programs that promote balanced online use. Policymakers and technology designers can also contribute to healthier usage patterns through interface modifications and awareness campaigns. Although the cross-sectional design and convenience sampling limit causal inference and generalizability, the large sample size and robust analytic approach strengthen the validity of the findings. Future research should prioritize longitudinal and cross-cultural studies to further clarify causal pathways and explore culturally adapted flourishing-enhancing interventions. Strengthening flourishing may represent a sustainable pathway for reducing vulnerability to PIU and supporting optimal mental health among digitally immersed emerging adults.

## Acknowledgements

 The authors gratefully acknowledge the participants for their valuable contribution to this study. The authors also thank Yaseen Ahmed for his valuable suggestions on the manuscript.

## Artificial Intelligence Use Disclosure

 During the preparation of this manuscript, the authors used DeepSeek V3.2 solely for language editing and for improving the clarity and readability of the text. The AI tool was used as a writing assistant to help rephrase sentences and restructure paragraphs for better flow, based on the authors’ original ideas, analysis, and data. After using this tool, the authors thoroughly reviewed and edited the content to ensure its accuracy, originality, and alignment with the intellectual contribution of the study. The authors take full responsibility for the final content of the manuscript. No AI tools were used to generate or interpret data or to replace the core intellectual work of the authors.

## Competing Interests

 On behalf of all authors, the corresponding author states that there is no conflict of interests.

## Ethical Approval

 The research was conducted according to the Declaration of Helsinki and relevant legal and ethical norms appropriate to the cultural context. The research protocol received formal ethical approval from the Institutional Review Board (IRB) of VIT-AP University prior to data collection (VIT-AP/IECI2025-26/007).

## Funding

 The authors did not receive support from any organization for the submitted work.

## References

[1] Statista. Internet and Social Media Users in the World 2025 [Internet]. Available from: https://www.statista.com/statistics/617136/digital-population-worldwide/?srsltid=AfmBOoqADKO48GpeXQDX_ZmlGRMejUwZR4zIintHYEtMlk2qI_zNhR8G. Accessed April 17, 2025.

[2] Metwally D, Bakari H, Manzoor A (2025). Social and psychological costs of problematic use of social media: users and gratification perspective. Cogent Psychol.

[3] Chemnad K, Aziz M, Belhaouari SB, Ali R (2023). The interplay between social media use and problematic internet usage: four behavioral patterns. Heliyon.

[4] Sujarwoto Sujarwoto, Saputri RAM, Yumarni T (2023). Social media addiction and mental health among university students during the COVID-19 pandemic in Indonesia. Int J Ment Health Addict.

[5] Prakash O. Is it time for India to set social media age limits for adolescents? Indian J Psychiatry 2025. 67(2):267-73. 10.4103/indianjpsychiatry.indianjpsychiatry_1012_24. PMC11964175 40181870

[6] Ahmed O, Walsh EI, Dawel A, Alateeq K, Espinoza Oyarce DA, Cherbuin N (2024). Social media use, mental health and sleep: A systematic review with meta-analyses. J Affect Disord.

[7] Gao J, Zheng P, Jia Y, Chen H, Mao Y, Chen S (2020). Mental health problems and social media exposure during COVID-19 outbreak. PLoS One.

[8] Sundarasen S, Chinna K, Kamaludin K, Nurunnabi M, Baloch GM, Khoshaim HB (2020). Psychological impact of COVID-19 and lockdown among university students in Malaysia: implications and policy recommendations. Int J Environ Res Public Health.

[9] Stănculescu E, Griffiths MD (2024). The association between problematic internet use and hedonic and eudaimonic well-being: a latent profile analysis. Technol Soc.

[10] Griffiths M (2005). A ‘components’ model of addiction within a biopsychosocial framework. J Subst Use.

[11] Van Rooij AJ, Prause N (2014). A critical review of “internet addiction” criteria with suggestions for the future. J Behav Addict.

[12] Ding H, Cao B, Sun Q (2023). The association between problematic internet use and social anxiety within adolescents and young adults: a systematic review and meta-analysis. Front Public Health.

[13] Tomska N, Rył A, Turoń-Skrzypińska A, Szylińska A, Marcinkowska J, Durys D (2022). Emotional state of young men in relation to problematic internet use. Int J Environ Res Public Health.

[14] Diotaiuti P, Mancone S, Corrado S, De Risio A, Cavicchiolo E, Girelli L (2022). Internet addiction in young adults: the role of impulsivity and codependency. Front Psychiatry.

[15] Gioia F, Rega V, Boursier V (2021). Problematic internet use and emotional dysregulation among young people: a literature review. Clin Neuropsychiatry.

[16] Pettorruso M, Valle S, Cavic E, Martinotti G, di Giannantonio M, Grant JE (2020). Problematic internet use (PIU), personality profiles and emotion dysregulation in a cohort of young adults: trajectories from risky behaviors to addiction. Psychiatry Res.

[17] Scott H, Woods HC (2018). Fear of missing out and sleep: cognitive behavioural factors in adolescents’ nighttime social media use. J Adolesc.

[18] Yu H (2024). Why do people use metaverse? A uses and gratification theory perspective. Telematics and Informatics.

[19] Ibáñez‐Sánchez S, Orus C, Flavian C (2022). Augmented reality filters on social media Analyzing the drivers of playability based on uses and gratifications theory. Psychol Mark.

[20] Katz E, Blumler JG, Gurevitch M (1973). Uses and gratifications research. Public Opin Q.

[21] Quan-Haase A, Young AL (2010). Uses and gratifications of social media: a comparison of facebook and instant messaging. Bull Sci Technol Soc.

[22] Mengistu N, Habtamu E, Kassaw C, Madoro D, Molla W, Wudneh A (2023). Problematic smartphone and social media use among undergraduate students during the COVID-19 pandemic: in the case of southern Ethiopia universities. PLoS One.

[23] Brand M, Wegmann E, Stark R, Müller A, Wölfling K, Robbins TW (2019). The Interaction of Person-Affect-Cognition-Execution (I-PACE) model for addictive behaviors: update, generalization to addictive behaviors beyond internet-use disorders, and specification of the process character of addictive behaviors. Neurosci Biobehav Rev.

[24] Seligman ME. Flourish: A Visionary New Understanding of Happiness and Well-Being. Simon & Schuster; 2011.

[25] Keyes CL (2005). Mental illness and/or mental health? Investigating axioms of the complete state model of health. J Consult Clin Psychol.

[26] Diener E, Wirtz D, Tov W, Kim-Prieto C, Choi DW, Oishi S (2010). New well-being measures: short scales to assess flourishing and positive and negative feelings. Soc Indic Res.

[27] VanderWeele TJ (2017). On the promotion of human flourishing. Proc Natl Acad Sci U S A.

[28] Butler J, Kern ML (2016). The PERMA-Profiler: a brief multidimensional measure of flourishing. Int J Wellbeing.

[29] Huppert FA, So TTC (2013). Flourishing across Europe: application of a new conceptual framework for defining well-being. Soc Indic Res.

[30] Crone EA, Konijn EA (2018). Media use and brain development during adolescence. Nat Commun.

[31] Arnett JJ (2000). Emerging adulthood A theory of development from the late teens through the twenties. Am Psychol.

[32] Keyes CLM. Atlanta: Brief Description of the Mental Health Continuum Short Form (MHC-SF) [Internet]. Atlanta, GA: Emory University; 2009. Available from: http://www.sociology.emory.edu/ckeyes. Accessed January 2025.

[33] Pramod SK, Raju S. Problematic Internet Use Questionnaire and Manual [dissertation]. Thiruvananthapuram: Department of Psychology, University of Kerala; 2008.

[34] Demetrovics Z, Szeredi B, Rózsa S (2008). The three-factor model of Internet addiction: the development of the Problematic Internet Use Questionnaire. Behav Res Methods.

[35] Kim HY (2013). Statistical notes for clinical researchers: assessing normal distribution (2) using skewness and kurtosis. Restor Dent Endod.

[36] O’Brien O, Sumich A, Baguley T, Kuss DJ (2023). A partial correlation network indicates links between wellbeing, loneliness, FOMO and problematic internet use in university students. Behav Inf Technol.

[37] Casale S, Lecchi S, Fioravanti G (2015). The association between psychological well-being and problematic use of internet communicative services among young people. J Psychol.

[38] Gür F, Gür GC (2025). The relationship between physical activity and problematic internet use in Turkish college students: the chain-mediated role of self-control and distress. Psychiatr Q.

[39] Webster D, Dunne L, Hunter R (2021). Association between social networks and subjective well-being in adolescents: a systematic review. Youth Soc.

[40] Bao T, Liang B, Riyanto YE. Social Media and Life Satisfaction: Evidence from Chinese Time-Use Survey. 2019. Available from: https://ssrn.com/abstract=3534633.

[41] Moreno MA, Jelenchick L, Cox E, Young H, Christakis DA (2011). Problematic internet use among US youth: a systematic review. Arch Pediatr Adolesc Med.

[42] Li S, Wang X, Wu Z, Zhang Y. The more internet access, the more mental symptoms students got, the more problematic internet use they suffered: a meta-analysis of mainland Chinese adolescents and young adults. Int J Ment Health Addict 2022:1-21. doi: 10.1007/s11469-022-00850-w. PMC9244201 35789813

[43] O’Keeffe GS, Clarke-Pearson K (2011). The impact of social media on children, adolescents, and families. Pediatrics.

